# Current views concerning the influences of murine hepatic endothelial adhesive and cytotoxic properties on interactions between metastatic tumor cells and the liver

**DOI:** 10.1186/1476-5926-4-8

**Published:** 2005-12-09

**Authors:** Hui Helen Wang, Hongming Qiu, Ke Qi, F William Orr

**Affiliations:** 1 Department of Health Sciences, Red River College and Department of Pathology, Faculty of Medicine, University of Manitoba, Winnipeg, Manitoba, Canada; 2 Department of Pathology, Health Sciences Center, University of Manitoba, Winnipeg, Manitoba, Canada; 3 Department of General Surgery, Nanshan Hospital, Shenzhen, Guangdong, China; 4 Department of Pathology, Faculty of Medicine, University of Manitoba, Winnipeg, Manitoba, Canada

## Abstract

Substantial recent experimental evidence has demonstrated the existence of reciprocal interactions between the microvascular bed of a specific organ and intravascular metastatic tumor cells through expression of adhesion molecules and nitric oxide release, resulting in a significant impact upon metastatic outcomes.

This review summarizes the current findings of adhesive and cytotoxic endothelial-tumor cell interactions in the liver, the inducibility, zonal distribution and sinusoidal structural influences on the hepatic endothelial regulatory functions, and the effects of these functions on the formation of liver cancer metastases. New insights into the traditional cancer metastatic cascade are also discussed.

## Introduction

The formation of a metastatic tumor in the secondary organ is the result of dissemination of a primary cancer cell, survival in the circulation, passing through the vascular bed in the distant organ and cancer cell proliferation [1-4]. Cancer metastasis is known to be an inefficient process, which reflects the fact that most of the intravascular cancer cells are killed within blood vessels or lymphatic channels [5,6]. Metastasis is accomplished in a step-wise or metachronous fashion [6,7]. More recent studies using mouse and rat models and *in vivo *video microscopy have demonstrated that the initial steps of the haematogenous metastatic process, from cancer cells entering the bloodstream to extravasating into secondary organs, are completed with remarkable efficiency [8,9]. The inefficiency is more associated with the subsequent steps involving cell division and formation of micrometastases by extravasated cancer cells in the secondary site [7,8,10]. In contrast, other studies have indicated that the majority of disseminating tumor cells die rapidly in the blood circulation and can not pass the first capillary bed they encounter [8,11-13]. With the metastatic cascade being well-outlined in the literature, the specific underlying mechanisms of tumor cell loss in the circulation and secondary organs, and the determinant factors for metastases formation still remain to be fully elucidated [3,10,14].

Recent *in vivo *and *in vitro *experimental evidence from various laboratories strongly suggests that, during the interactions between an organ microvascular bed and intravascular tumor cells, nitric oxide (NO) plays a significant role as a cytotoxic natural defensive effector, produced by the vascular endothelial cells, to exert toxic effects on invading tumor cells, interact with endothelial adhesion molecules and regulate the subsequent metastatic tumor formation in the secondary organ [10,15,16]. This review surveys this new evidence and reviews current opinions derived mostly from animal studies on how endothelial and tumor cells interact with each other through adhesive and cytotoxic properties in the hepatic microvascular bed. We describe how these interactions and metastases formation can be influenced by sinusoidal structural and functional characteristics and alterations. The identification of this host internal defensive mechanism gives new insights into cancer metastatic inefficiency, and identifies a new barrier in the classic model of the cancer metastatic cascade.

## Influence of hepatic adhesive properties

### Endothelial-tumor cell interactions are regulated by inducible adhesion molecules expressed in the liver

Since the "seed and soil" theory proposed by Stephen Paget, there has been a long history of research into the reasons for organ-specific cancer metastasis [17,18]. The liver is a common site for metastasis of human cancer and a convenient target for experimental studies of metastasis. From the latter it is apparent that endothelial cell surface adhesion molecules have an extensive role in regulating cancer cell site-specific arrest, transendothelial migration and metastases formation [3,19-23].

Expression of various hepatic endothelial adhesion molecules has been demonstrated to be selectively inducible by cytokines, bacterial lipopolysaccharide (LPS) or arresting tumor cells in the liver microvascular bed. In turn, these adhesion molecules can be shown to regulate the arrest of circulating cancer cells in the hepatic sinusoids. For example, interleukin-1α (IL-1α) pretreatment of mice altered the melanoma cell (B16F1) arrest pattern from 32 μm beyond the sinusoidal inlet to larger terminal portal venules (TPV) observed by intravital videomicroscopy, suggesting increased adhesive interactions between endothelial and tumor cells following IL-1α stimulation [24]. Interleukin-18 (IL-18) has been demonstrated *in vivo *and *in vitro *to promote liver metastasis by enhancing melanoma cell adhesion to the hepatic sinusoidal endothelial cells via microvascular VCAM-1 (vascular cell adhesion molecule-1) expression [25-27]. With a basal expression level of ICAM-1 (intercellular adhesion molecule-1), minimal expression of VCAM-1 and no expression of E-selectin or αv integrin in unstimulated mouse livers, 1 μg/g body weight of LPS i.p. selectively induced the expression of ICAM-1 (4–48 h), VCAM-1 (4–24 h) and E-selectin (2 h) on the sinusoidal lining cell surface, while αv integrin expression was unchanged [28,29]. LPS did not significantly alter the expression of VLA-4 (very late antigen-4, counter receptor of VCAM-1) or LFA-1 (leukocyte functional antigen-1, counter receptor of ICAM-1) on melanoma cells either *in vivo *or *in vitro *[30]. Tumor necrosis factor (TNF) induced sustained VCAM-1 expression within 4 h in the lung, liver and kidney of mice [31], and increased the adhesion of highly metastatic murine carcinoma cell line H-59, and human colorectal carcinoma lines HM 7 and CX-1 to murine hepatic endothelial cells in the primary culture. This effect was completely abolished by a monoclonal antibody to murine E-selectin [21]. Mannose receptor-mediated endothelial cell activation also contributed to B16 melanoma cell adhesion and metastasis in the mouse liver [32].

The expression of sinusoidal adhesion molecules is affected by metastatic cells in the hepatic microenvironment. The arrest of B16F1 melanoma cells in the liver sinusoids (following mesenteric vein injection) induced focal expression of VCAM-1 and more diffuse expression of ICAM-1 around the melanoma cell arrest sites [30]. Similarly, the arrest of murine carcinoma line H-59 cells after intrasplenic injection induced E-selectin expression on the hepatic sinusoidal endothelium between 2–24 h [33]. The expression of ICAM-1, VCAM-1, E-selectin and αv integrin was all induced to different degrees by the growth of melanoma tumors in the peritoneal cavity without liver metastasis in the mouse [28]. In a study on progression of mouse melanoma (B16-BL6) spontaneous metastasis, organ specific induction of VCAM-1 was observed in the cardiac, hepatic and cerebral vascular beds 4 weeks following the resection of primary tumors when metastatic pulmonary burden was maximal [34]. Intrasplenically injected B16 melanoma (B16M) cells also increased the expression of VCAM-1 significantly on hepatic sinusoidal endothelial cells within the first 24 h, which correlated with the increased *in vitro *adhesion of B16M cells to hepatic sinusoidal endothelial cells isolated from B16M cell-injected mice [35].

The mechanisms and significance of the selectivity of adhesion molecule induction have not been fully described at this stage. However, the inducibility of various adhesion molecules on the hepatic endothelial cell surface by different microenvironmental stimuli has provided a potential diversity and flexibility for sinusoidal endothelial cells to participate in tumor defensive responses when intravascular metastatic cancer cells are present.

### Impact of sinusoidal structural and functional characteristics on adhesion molecule induction

The micro-structural and functional heterogeneity in the liver across its functional unit of acinar zonation has been well-described in the literature [36-39]. This hepatic zonal heterogeneity has also played a significant role influencing the patterns of induced adhesion molecule expression. Differential zonal expressions of certain adhesion molecules induced by LPS stimulation have been demonstrated [28]. With a weak expression around the terminal portal venule regions (acinar zone 1) under basal conditions, ICAM-1 was induced to a uniform strong expression (4–48 h) across each entire acinus in the liver following LPS administration. On the contrary, VCAM-1 and E-selectin both had minimal or no expression in unstimulated livers, but had significantly stronger expression in acinar zone 1 than zone 2 and 3 after LPS stimulation, with VCAM-1 expressed between 4–48 h and E-selectin 2–12 h [28]. LPS stimulation also increased the retention of B16F1 melanoma cells in the liver between 8–24 h, especially in the terminal portal venule region presumably through increased expression of adhesion molecules, ICAM-1 and VCAM-1 [30]. IL-1 zonal heterogeneity of mannose receptor-mediated ligand endocytosis in the mouse and rat liver was also observed using flow cytometry following LPS stimulation [40,41]. In human studies, major differences have been noted in the composition of the portal tract and sinusoid with regard to endothelial and parenchymal cell expression of cell-cell and cell-matrix adhesion molecules during inflammatory reactions in human liver grafts [42]. Differential expression of various adhesion molecules has been reported between normal and inflamed livers, or livers rejected after transplantation in humans. The selectins ELAM-1 (endothelial leukocyte adhesion molecule) and CD62 (cluster of differentiation 62) were basally expressed and inducible on portal tract endothelia and central vein endothelia with acute and chronic human liver inflammation, although sinusoidal endothelia lack this mechanism even with severe inflammation [43]. Portal and sinusoidal endothelia showed a different expression and inducibility of VCAM-1, ICAM-1, ICAM-2, and LFA-3 (leukocyte functional antigen-3) in human livers [43].

In addition to the impact of hepatic zonal heterogeneity on adhesion molecule expression, alterations in the liver sinusoidal architecture also significantly change the endothelial cell surface molecule expression and tumor cell behavior patterns. Using a murine liver cirrhosis model, where the sinusoidal lumens were narrowed due to the formation of fibrous tissue, the expression of adhesion molecules ICAM-1 and VCAM-1 was found to be significantly increased (stronger in acinar zone 1) on the endothelial surface with E-selectin undetectable [44]. After injecting melanoma cells into the portal vein, melanoma cell retention in the cirrhotic liver terminal portal venule regions was also significantly increased in comparison with the control livers [44].

## Influence of hepatic cytotoxic properties

Recent experimental evidence suggests that in addition to adhesion molecules, the hepatic sinusoid has other heterogeneous structural and functional properties that create a unique anatomical vascular bed in which endothelial lining cells exert antitumor effects with extensive diversity and flexibility to fight against invading metastatic tumor cells.

### Endothelial-tumor cell interactions induce nitric oxide release from the hepatic endothelium

Direct and indirect evidence from the literature has supported the hypothesis that the hepatic sinusoidal microvasculature is toxic to metastatic tumor cells. Various experimental data obtained to date have indicated that the hepatic endothelium exerts its antitumor defensive effects through the release of NO and other reactive oxygen species (ROS) [4,10,15,16,45-47]. As with adhesion molecule expression, the cytotoxic regulatory functions in the liver have also been demonstrated to be inducible by microenvironmental stimuli.

**Figure 1:**
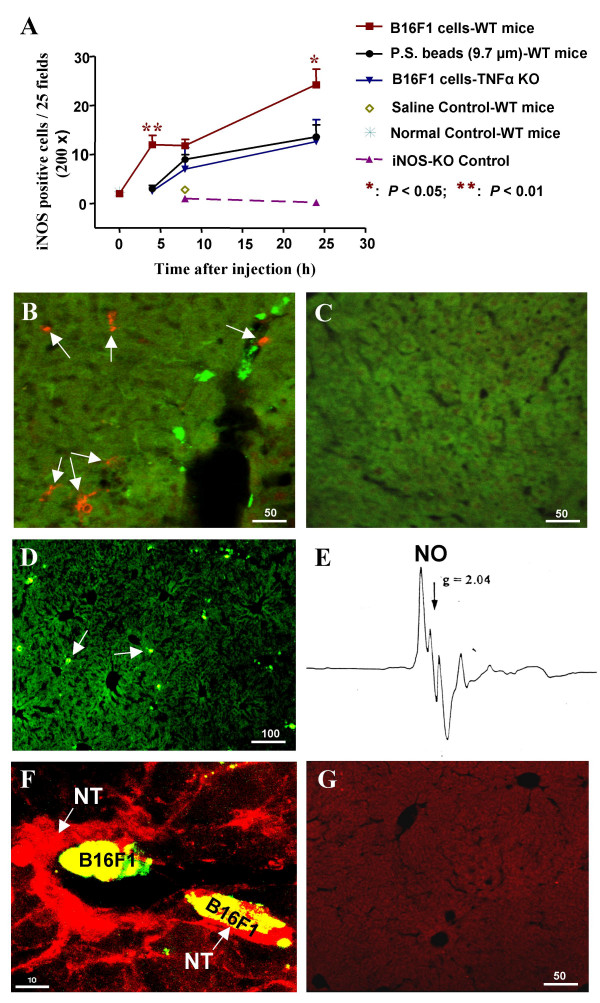
**B16F1 melanoma cell-induced iNOS expression, NO production and nitrotyrosine formation in the mouse liver**. (A): Induction of hepatic iNOS expression (0–24 h) in various strains of C57BL/6 mice injected with B16F1 melanoma cells or polystyrene (P.S.) beads. iNOS was detected by immunofluorescent double-labeling using rabbit anti-mouse iNOS as the primary and Cy™3-conjugated goat anti-rabbit IgG as secondary antibodies. Data represent the mean ± SE of iNOS positive cells in 25 fields of each mouse liver in the group (n = 5 mice/group, at 200 × magnifications). WT: Wild-type. KO: Knockout; (B): iNOS expression (orange, arrows) in sinusoidal lining cells and hepatocytes of a wild-type mouse liver at 24 h after injection of melanoma cells (green); (C): iNOS detection negative control in a normal wild-type liver without cell injection. (D): Liver sample excised immediately after B16F1 cell injection (arrows, 0 h, 100 ×); (E): NO signal detected in the 0 h liver sample using EPR spectroscopy; (F): Nitrotyrosine (NT, red, arrows) detection in the same 0 h liver, by double-labeling immunohistochemistry using mouse anti-nitrotyrosine primary antibody, along the sinusoidal wall adjacent and inside the arresting tumor cells. (G): A negative control of NT detection in a wild-type mouse liver without tumor cell injection. Scale bars displayed in μm.

The original evidence that hepatic endothelium-derived NO is induced by intravascular metastatic tumor cells was obtained by applying electron paramagnetic resonance (EPR) NO-spin trapping technologies into a classic murine melanoma metastatic model [15,48,49]. By injecting fluorescent microsphere-labeled B16F1 melanoma cells into the portal circulation of C57BL/6 mice, a swift burst of NO was detected in liver samples within 5 minutes of cell injection. NO induced apoptosis in 20–30 % of the melanoma cells arresting in the liver after 4 h [15]. NO was identified and its cytotoxicity to melanoma cells was supported by finding that the nonselective NO synthase inhibitor L-NAME (N_G_-nitro-L-arginine methyl ester) blocked NO production and melanoma cell apoptosis in the sinusoids. The ability of a short burst of NO to cause apoptosis was further confirmed by detecting the apoptotic DNA fragmentation and cell membrane damage in B16F1 cells exposed to a NO donor for 5 min *in vitro *[15]. The mechanisms of tumor cell specific induction of NO release at the site of cell arrest have not yet been identified but are suggested to be partly due to tumor cell-induced vascular wall shear stress with circumferential stretch and isometric contraction (Lower levels of NO are released following injection of inert microspheres with similar diameters to melanoma cells) [15,50,51]. Using an *in situ *liver perfusion system, the cellular origin of the NO release following B16F1 cell arrest in the liver has been identified as periportal endothelial and sinusoidal lining cells, and hepatocytes adjacent to the arresting B16F1 cells [52]. The endothelial and sinusoidal lining cells released NO in an eNOS (endothelial NO synthase)-dependent manner over a time of 500 sec, and hepatocytes over a longer period of time measured by fluorescent 4,5-diaminofluorescein diacetate (DAF-2 DA) used as the NO detection probe [52]. In addition to the immediate burst of endothelial eNOS-dependent NO production upon melanoma cell arrest in the liver, a delayed iNOS (inducible NO synthase)-dependent cytotoxic NO induction after 4 h of cell injection into the mesenteric vein has also been demonstrated, which was partially due to the shear forces generated by melanoma cell arrest in the sinusoids, and produced from both sinusoidal lining cells and hepatocytes detected by double-labeling immunohistochemistry [15] (Figure 1: A – C).

This evidence has been supported by findings from Umansky *et al*. [4,16] and Rocha *et al*. [53]. Using a well-characterized ESbL-lacZ mouse T lymphoma model, the authors have shown that a significant increase in NO production detected *in vitro *from *ex vivo *isolated liver endothelial cells and Kupffer cells coincided with the plateau phase (tumor retardation phase) of primary tumor growth and a low level of liver metastasis. They have also demonstrated that the activated host liver endothelial cells play dual roles in metastatic processes by expressing adhesion molecules and producing NO from iNOS activation [4,16,53].

Edmiston *et al*. have shown that unstimulated murine sinusoidal endothelial cells produced ROS that were selectively toxic to weakly metastatic human colorectal carcinoma clone A cells, with the toxicity blockable by pretreatment with NO synthase inhibitor, superoxide dismutase or dexamethasone [54]. Coculture of ischemic liver fragments with human colorectal carcinoma cells killed more weakly metastatic clone A cells at 24 h than highly metastatic CX-1 cells because of the higher sensitivity to NO and ROS in clone A cells [47,55]. NO also induced apoptosis in different human neoplastic lymphoid cells and breast cancer cell lines through caspase activation pathways [46,56].

### Endothelial-tumor cell interactions induce release of other inducible reactive oxygen species (ROS) from the hepatic endothelium

In addition to NO, other cytotoxic ROSs are released from the liver endothelium and also possess antitumor cytotoxicity. *In vitro*, sinusoidal endothelial cells release hydrogen peroxide (H_2_O_2_) which enhanced VLA-4 mediated melanoma cell adherence to the hepatic sinusoidal endothelium and caused tumor cytotoxicity after IL-1 treatment in mice [57,58]. Superoxide anion (O_2_^-^) may be involved in the cytotoxicity of murine hepatic sinusoidal endothelial cells to weakly metastatic human colorectal carcinoma cells [47,54,55]. The important interplays between NO and other ROSs, such as O_2_^-^, in cancer development and progression have been reviewed [45]. The rapid death of most cancer cells after delivery to some target organs has also been demonstrated to be a consequence of their mechanical interactions within the microvasculature [12].

The accumulated evidence to date has directed us to recognize the existence of a host natural defensive mechanism network in the hepatic microvasculature through the production of NO and other ROSs from the sinusoidal endothelium to generate cytotoxicity to invading intravascular tumor cells to fight against cancer metastasis in the liver.

### Impact of sinusoidal structural and functional characteristics on nitric oxide induction

Similar to the inducible adhesion molecule expression under the influence of hepatic zonal heterogeneity, evidence suggests that the release of NO from the hepatic endothelium is restricted to specific anatomical zones. Using an *in situ *C57BL/6 mouse liver perfusion system, the levels of NO production without and with tumor cells in the liver were found to be much greater in acinar zone 1 than zone 2 and 3 by direct visualization of NO synthesis through deesterification and conversion of intracellular DAF-2 DA to DAF-2T [52]. In cirrhotic mouse livers with altered sinusoidal architecture, significantly lower levels of NO production were detected both under basal conditions (without tumor cells) and after tumor cell arrest by the same experimental system [44].

### Evidence of cytotoxic properties in extrahepatic microvascular beds

The detection of a host defensive mechanism existing in the hepatic endothelium has raised the question of whether similar defense mechanisms also exist in other metastatic target organs. Direct *in vitro *lysis of metastatic tumor cells by cytokine-activated murine lung vascular endothelial cells has been demonstrated. NO (detected by nitrite concentration in the culture medium) produced by interferon gamma and TNF-activated lung vascular endothelial cells played a major role in the lytic destruction of reticulum cell sarcoma [59,60]. Rapid death of transformed metastatic rat embryo cells, occurred via apoptosis in the lungs 24–48 h after injection into the circulation of immune-deficient nu/nu mice, has been reported [14,61].

Using EPR NO-spin trapping technologies, a significantly increased production of NO was detected in lung tissue samples between 20 min and 4 h after the tail vein injection of fluorescent microsphere-labeled B16F1 melanoma cells [49]. The EPR results were also supported in an isolated, ventilated and blood-free mouse lung perfusion model, where NO production *in situ *was observed in real time using intact organ microscopy techniques. Fluorescent NO signals (DAF-2T) increased rapidly at the site of tumor cell arrest in the lungs and continued to increase throughout 20 min thereafter [49,62]. NO contributed to tumor cell apoptosis since 3-fold more B16F1 cells subsequently underwent apoptosis in the lungs of wild-type mice compared to animals in which NO production was inhibited, in particular, in eNOS-deficient mice and NOS inhibitor L-NAME-pretreated mice [49].

The identification of a similar antitumor defensive mechanism in the pulmonary microvascular bed has reinforced the concept that the host can release NO and other ROSs as cytotoxic effector molecules to fight against the invading metastatic tumor cells in the microvascular beds of the first-line cancer metastatic organs, such as the liver and lung.

### Molecular mechanisms of nitric oxide-induced melanoma cell cytotoxicity

The majority of reports indicate that the underlying molecular mechanisms for NO-induced tumor cell cytotoxicity are direct damage to DNA and the cell membrane, or activation of apoptosis-initiating caspases (cysteine proteases) causing tumor cell apoptosis and necrosis [10,14,15,45-47,49,54,56,59,60]. In addition, preliminary evidence also suggests that NO may induce oxidative damage on proteins through NO-superoxide-peroxynitrite and NO-nitrogen dioxide-nitrite pathways to form nitrotyrosine. The latter is the footprint of potent short-lived reactive nitrogen species, peroxynitrite (ONOO^-^) production *in vivo*, mediating NO-induced oxidative attacks on biological macromolecules [63-65] (Figure 1: D–G). More observations need to be made to provide supportive evidence along this direction.

### Interactions between nitric oxide and adhesion molecules in the hepatic microvascular bed

The importance of interplays between NO and adhesion molecules in the regulation of liver cancer metastasis has been recognized and addressed in recent years [10,19,20,45]. The inducible murine hepatic microvascular adhesive and cytotoxic regulatory functions have been regulated by using LPS [28,30]. With enhanced local expression of VCAM-1 and ICAM-1 around B16F1 cell arrest sites in the liver, LPS significantly increased the retention of melanoma cells in the liver, especially in the terminal portal venule regions between 8 and 24 h after intramesenteric injection of melanoma cells [30]. LPS also significantly increased the levels of iNOS expression and tumor cell induced-NO production at 8 h after administration and cell injection, and increased the rates of B16F1 cell apoptosis in the terminal portal venule region [30]. These data have been interpreted to indicate that LPS stimulated a synergistic interaction by inducing both the hepatic endothelial adhesion molecule expression and NO release in the terminal portal venular regions, resulting in higher levels of tumor cell killing in this region in the liver [30]. The dual roles of activated host liver endothelial cells in murine lymphoma metastatic process have also been reviewed [16]. On one hand, upregulation of the expression of particular adhesion molecules is considered to lead to the increased tumor cell binding and stimulation of angiogenesis, and on the other hand, endothelial cells can contribute to host anti-metastatic responses by producing the cytotoxic molecule NO from arginine with the help of iNOS [16]. Synergistic interactions between LFA-1/ICAM-1 and lymphoma progression phases with cytotoxic NO production have been described [4]. Interactions between cytokine IL-18, VCAM-1, H_2_O_2 _and hepatic sinusoidal endothelial cells have also been demonstrated [35]. Recombinant catalase administered *in vivo *completely blocked the increase of VCAM-1 expression induced by B16M cell arrest in the liver, and blocked *in vitro *B16M cell adhesion to sinusoidal lining cells isolated from B16M cell-injected mice [35]. Incubation of hepatic endothelial cells with nontoxic concentrations of H_2_O_2 _directly enhanced VCAM-1-dependent B16M cell adhesion *in vitro *without proinflammatory cytokine mediation [35].

In addition to synergistic interactions between NO and adhesion molecules, their counteractive interactions have also been identified. NO reduces tumor cell adhesion to isolated rat postcapillary venules *in vitro *[66]. Anti-adhesive roles of constitutively produced NO in inhibiting leukocyte rolling and adhesion in the microcirculation have been described [67,68]. Oxidative stress in the liver can be caused by ischemia/reperfusion (I/R) injury when tumor cells entering the hepatic microcirculation obstruct hepatic sinusoids and temporarily occlude blood flow before the hepatic circulation is reestablished by either tumor cell death or invasion into the parenchyma [8,55]. The counteractive roles of NO with adhesion molecules, such as decreasing P-selectin and ICAM-1 mRNA expression, attenuating neutrophil accumulation and liver damage in hepatic ischemia/reperfusion injury have been reviewed [69-71]. IL-10 has also been shown to inhibit hepatic I/R injury by inhibiting the upregulation of iNOS expression following I/R injury [55]. The multifaceted roles and effects of NO and adhesion molecule interactions support the scenario that the host uses this flexible natural defensive mechanism to protect itself from a variety of disastrous oxidative injuries and tissue damages to the hepatic microvasculature.

## Effects of sinusoidal adhesive and cytotoxic functions on metastasis

Substantial experimental evidence supports the hypothesis that hepatic adhesive functions can regulate cancer metastatic outcomes in the liver. IL-1α pretreated mice had 11-22-fold greater hepatic melanoma tumor burden than control mice pretreated with saline presumably through altering adhesive interactions between B16F1 cells and the hepatic microvasculature [24,72]. Liver sections from IL-1α-pretreated mice attracted 3-fold more melanoma cells to adhere *in vitro *than control liver sections. Adhesion was blocked by antibodies to E-selectin, ICAM-1, VCAM-1 and αv integrin subunit [24]. A single dose of IL-1 receptor antagonist (0.2 mg/kg, i.p.) given 2 h before intrasplenic injection of melanoma cells reduced the number of hepatic metastases by 50% and metastatic volume by 70% compared with the vehicle-injected control mice [73]. Systemic inflammation induced by intravenous injection of IL-1 or LPS increased hepatic melanoma metastasis significantly in an IL-1 dependent manner [74]. E-selectin expression blockage by monoclonal antibody significantly reduced experimental liver metastasis in the mouse [21]. Blockade of VCAM-1 expression *in vivo *with specific antibodies, administered before B16M cell injection into the portal circulation, decreased sinusoidal retention of luciferase-transfected B16M cells by 85%, and metastasis development by 75%, indicating that VCAM-1 expression on tumor-activated sinusoidal endothelial cells had a prometastatic contribution [35].

In addition to such adhesive functions, hepatic cytotoxic properties alone or through interactions with adhesive function and hepatic vascular zonal heterogeneity have been demonstrated to contribute significantly to the inhibition of tumor growth in the secondary sites. With 2/3 of intramesenteric injected-B16F1 cells arresting in the liver sinusoids, the rapid burst of NO induction triggered apoptosis in 1/4 of the intravascular melanoma cells and significantly decreased the metastatic tumor burden in the liver [15]. Increased NO production by *ex vivo *isolated liver sinusoidal endothelial cells was detected in the tumor growth retardation phase in a well-characterized murine T lymphoma model, and the breakdown of this NO synthesis coincided with the second tumor expansion phase [4,16,53]. LPS has been demonstrated to inhibit melanoma metastases formation in the liver by inducing NO release and adhesion molecule expression in the hepatic endothelium, which was primarily located within the terminal portal venular region (acinar zone 1) [30]. Selective implantation and growth in rats and mice of experimental liver metastasis in acinar zone 1 has been demonstrated using B16 melanoma and Lewis lung carcinoma cell lines [75]. Cirrhotic livers with narrowed sinusoidal lumens were found to have decreased velocity of melanoma cell traveling in the sinusoids, decreased NO release and tumor cell apoptosis, and increased tumor cell proliferation and metastases formation in the liver [44]. The vascular-targeting agent ZD6126 was able to reduce the liver metastatic burden significantly in mice with extensive tumor necrosis, increased tumor cell apoptosis and a reduction in tumor-associated vasculature with disrupted and non-functional vascular channels within metastases with no blood flow [76]. In the pulmonary vascular bed, NO production following tail vein injection of B16F1 melanoma cells induced 3-fold higher apoptosis rate, 30 % higher tumor cell clearance, and 2 to 5-fold less metastases formation in wild-type mice in comparison with the controls [49].

Given the functional and structural features (adhesion, cytotoxicity, zonal differentiation) of the hepatic microvasculature, and the fact that the liver and lung are the most common metastatic target organs, the ability of their vasculatures to produce cytotoxic molecules is of considerable interest as a means to protect the host from circulating metastatic cells. The presence of a tumor-killing defensive mechanism in the liver and lungs provides an additional explanation for tumor cell loss in these secondary organs and helps to explain the inefficient process of cancer metastasis.

## Cancer metastatic cascade modified

**Figure 2:**
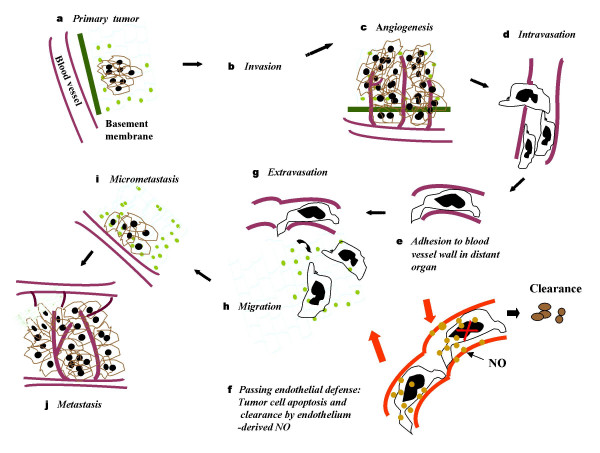
**Modified classic metastatic cascade**. Traditional metastatic cascade: Steps a → b → c → d → e, → g → h → i → j. Modified metastatic cascade: Steps a → b → c → d → e → **f **→ g → h → i → j, with a new "**f**" step of passing through endothelial defense mechanisms included.

The compelling data elaborated above on regulations of liver cancer metastasis by the hepatic microvascular adhesive and cytotoxic functions prompted us to review the classic metastatic cascade again, which includes the primary tumor cell local invasion, intravasation, circulation, adhesion and extravasation, survival and proliferation in the secondary organ [3,4,8,22,77]. A new step in which tumor cells pass through the host endothelial defensive mechanisms has been incorporated into the traditional model (Figure 2).

## Conclusion

In summary, there is convincing evidence that hepatic endothelial adhesive and cytotoxic properties can significantly influence the interactions between metastatic tumor cells and the liver with a consequence of altering the formation of liver metastases. In addition, the hepatic endothelial adhesive and cytotoxic functions are inducible, zonal, heterogeneous, affected by sinusoidal structural alterations, and can interact with each other synergistically or counteractively. Together they provide the liver with a specific vascular bed with extensive diversity and flexibility to fight against invading metastatic tumor cells and other tissue injuries. A similar inducible antitumor defensive mechanism also exists in the pulmonary microvascular bed. The molecular mechanisms of the hepatic endothelial cytotoxicity are beginning to be identified. Production of NO and other ROSs from the sinusoidal endothelium causes damage to tumor cell DNA, cell membrane, and protein macromolecules. This natural defensive mechanism in the hepatic and pulmonary microvasculature contributes to our understanding of tumor cell loss in the secondary organ, helps to explain cancer metastatic inefficiency, and is an additional barrier to metastasis in the classic model of the cancer metastatic cascade.

## Competing interests

The author(s) declare that they have no competing interests.

## Authors' contributions

HHW performed the work on hepatic adhesion molecule expression, NO cytotoxicity to tumor cells, regulation of cancer metastasis by LPS stimulation, and wrote the review manuscript. HQ performed the work on NO cytotoxicity in the lungs, iNOS induction by arresting tumor cells in the liver, NO detection by DAF-2 DA and participated in the manuscript revision. QK performed the work on visualizing NO production in the liver by DAF-2 DA, melanoma metastasis in cirrhotic livers and participated in the manuscript revision. FWO was the supervisor of all studies, publications and performed the revision of the review. All authors have read and approved the final manuscript.
